# Cell cycle reentry triggers hyperploidization and synaptic dysfunction followed by delayed cell death in differentiated cortical neurons

**DOI:** 10.1038/s41598-018-32708-4

**Published:** 2018-09-25

**Authors:** E. Barrio-Alonso, A. Hernández-Vivanco, C. C. Walton, G. Perea, J. M. Frade

**Affiliations:** 1 Department of Molecular, Cellular and Developmental Neurobiology, Cajal Institute (CSIC), Madrid, Spain; 2 Department of Functional and Systems Neurobiology, Cajal Institute (CSIC), Madrid, Spain

## Abstract

Cell cycle reentry followed by neuronal hyperploidy and synaptic failure are two early hallmarks of Alzheimer’s disease (AD), however their functional connection remains unexplored. To address this question, we induced cell cycle reentry in cultured cortical neurons by expressing SV40 large T antigen. Cell cycle reentry was followed by hyperploidy in ~70% of cortical neurons, and led to progressive axon initial segment loss and reduced density of dendritic PSD-95 puncta, which correlated with diminished spike generation and reduced spontaneous synaptic activity. This manipulation also resulted in delayed cell death, as previously observed in AD-affected hyperploid neurons. Membrane depolarization by high extracellular potassium maintained PSD-95 puncta density and partially rescued both spontaneous synaptic activity and cell death, while spike generation remained blocked. This suggests that AD-associated hyperploid neurons can be sustained *in vivo* if integrated in active neuronal circuits whilst promoting synaptic dysfunction. Thus, cell cycle reentry might contribute to cognitive impairment in early stages of AD and neuronal death susceptibility at late stages.

## Introduction

Alzheimer’s disease (AD), the most common cause of dementia, is an irreversible neurological disorder characterized by progressive cognitive decline and degeneration of brain regions crucial for learning and memory^1^. One of the earliest cellular processes observed in the AD brain is cell cycle reentry in neurons^2^. Work performed during the last two decades has revealed that cell cycle reentry may be abortive, triggering neuronal cell death at the G1/S checkpoint^3^, or non-abortive, leading to DNA synthesis followed by cell death before undergoing G2/M transition^4^. In AD, most neurons that reactivate the cell cycle undergo DNA synthesis and remain with hyperploid DNA content (i.e. above 2 C)^5–7^ until later stages of the disease, when they specifically undergo delayed cell death^5,8–10^. Cell cycle reentry in these neurons could lead to functional alterations underlying the etiology of AD^11^. In this regard, we have recently demonstrated that age-associated, neuronal tetraploidization correlates with reduced cognitive capacity in mice^7^. Unfortunately, the physiological changes occurring in neurons that undergo cell cycle reentry and become hyperploid remain unknown due to the lack of molecular markers to identify these cells *in vivo*. An alternative approach for the analysis of these changes would be the use of neurons that are forced to reactivate the cell cycle.

Forced cell cycle reentry in postmitotic neurons can be achieved by SV40 large T antigen (TAg) expression^12,13^, a manipulation that triggers neuronal degeneration in different neural tissues^12–15^. Neuronal expression of TAg *in vivo* recapitulates the hallmarks of AD, including the presence of neurofibrillary tangle-like profiles and plaque-like amyloid deposits^13^. In this latter study, TAg was widely expressed in neurons, resulting in widespread neuronal cell cycle reentry. This situation differs from AD, a condition characterized by a small proportion of neurons becoming hyperploid^5–7^, which remains surrounded by non-affected neurons.

To study the functional changes triggered by cell cycle reentry in a restricted population of differentiated neurons we have used cortical cultures lipofected with TAg. This protocol, which results in ~1% transfection efficiency, affords the characterization of the hyperploidization process and allows the study of the functional changes occurring in neurons that reactivate the cell cycle while connected with diploid neurons, as occurs in AD. We have focused on the synaptic function in these cells, as synaptic failure is known to be an early feature of AD^16^, preceding neuronal degeneration^17^ and correlating with cognitive impairment^18^.

Here we report that ~70% of transfected cortical neurons, which reactivate the cell cycle in response to TAg expression, become hyperploid. We also show that cell cycle reentry specifically triggers synaptic dysfunction in cortical neurons, which correlates with reduced expression in these cells of the postsynaptic scaffold protein PSD-95 and impairment of the axon initial segment (AIS), a specialized membrane region that sustains neuronal polarity and integrates synaptic input to generate action potentials^19^. TAg-expressing neurons initially survive, but cell cycle reentry specifically and progressively triggers non-apoptotic/oxidative stress-independent death. Finally, we provide evidence that facilitating membrane depolarization after addition of high extracellular potassium prevents further loss of PSD-95 puncta and partially restores spontaneous activity in neurons that reactivate the cell cycle, which is concomitant with survival facilitation.

## Results

### TAg expression induces DNA synthesis and hyperploidy in most cortical neurons

**Figure 1:**
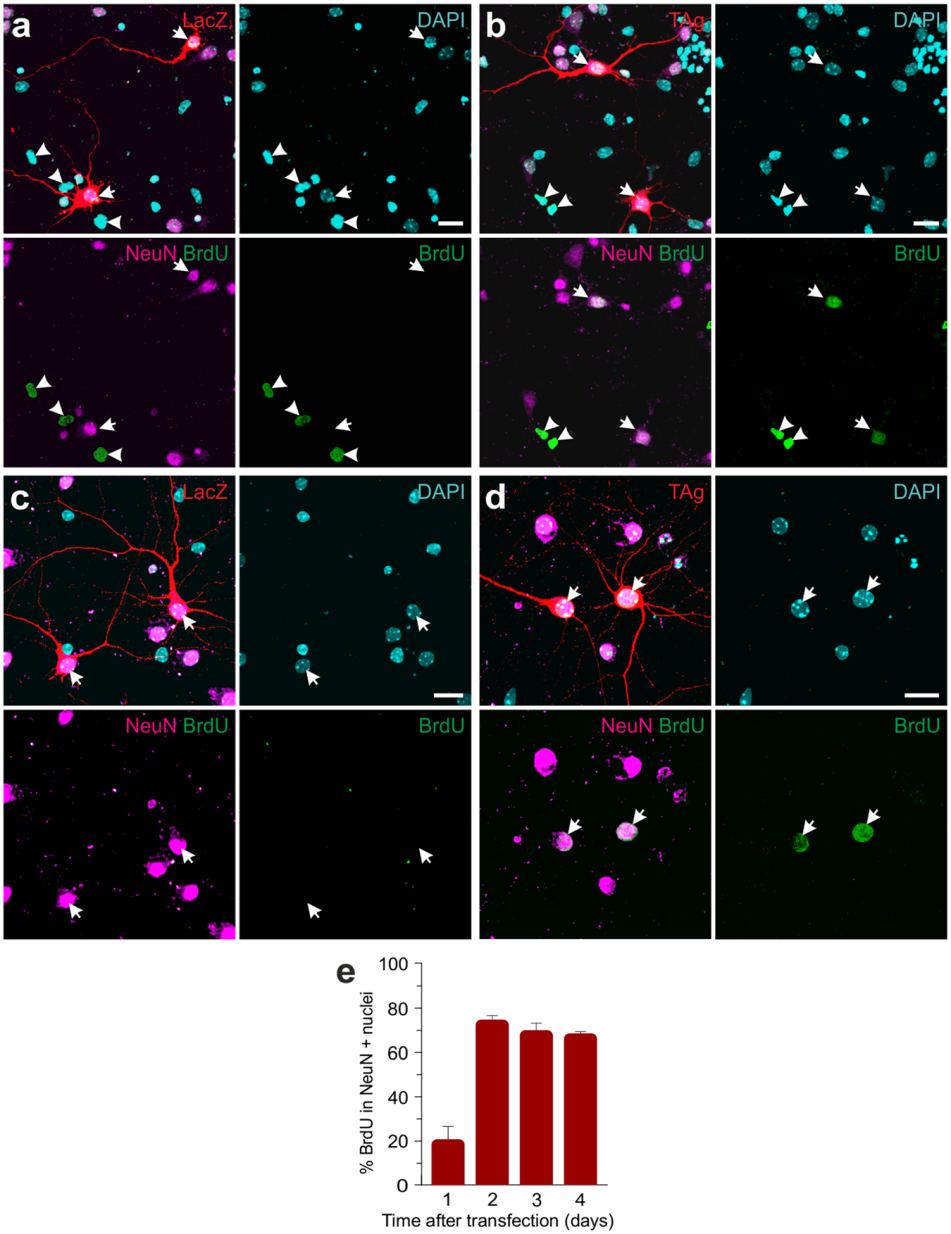
TAg expression induces cell cycle reentry in cortical neurons. (**a**) RFP/LacZ-transfected cortical neurons (arrows) after 1 dpt. LacZ-expressing (red)/NeuN-positive neurons (purple) do not incorporate BrdU (green). In contrast, LacZ-expressing/NeuN-negative cells incorporate BrdU (arrowheads). (**b**) RFP/TAg-transfected cortical neurons (arrows) after 1 dpt. TAg-expressing (red)/NeuN-positive neurons (purple) incorporate BrdU (green). DNA is labeled with DAPI (blue). LacZ-expressing/NeuN-negative cells also incorporate BrdU (arrowheads). (**c**) RFP/LacZ-transfected cortical neurons (arrows) after 2 dpt. LacZ-expressing (red)/NeuN-positive neurons (purple) do not incorporate BrdU (green). (**d**) RFP/TAg-transfected cortical neurons (arrows) after 2 dpt. TAg-expressing (red)/NeuN-positive neurons (purple) incorporate BrdU (green). DNA is labeled with DAPI (blue). (**e**) Percentage of RFP/TAg-transfected cortical neurons incorporating BrdU at the indicated time points. Confocal projection images are shown in (**a**–**d**). Error bars indicate SEM (n = 3). Bars: 20 μm.

To confirm that TAg expression can trigger neuronal cell cycle reentry, cortical neurons maintained for 6–8 days *in vitro* (DIV) were lipofected with RFP and either TAg or LacZ and then treated with BrdU, a nucleoside analog that becomes incorporated into the DNA during S-phase. Cultures were fixed at different time points after transfection and subjected to double immunostaining with antibodies against NeuN, a well characterized neuronal marker^20^, and BrdU. Then, the proportion of BrdU incorporation was evaluated in living NeuN-positive neurons. Transfected neurons were identified by the expression of RFP. We confirmed in TAg/RFP transfected cultures that all RFP-positive neurons analyzed show TAg-specific immunostaining (102 RFP-positive/TAg-positive neurons, 0 RFP-positive/TAg-negative neurons, and 3 RFP-negative/TAg-positive neurons were detected) (Fig. S1). BrdU immunostaining indicated that control neurons (i.e. LacZ-transfected NeuN-positive cells) did not incorporate this nucleotide analogue at any time point (Fig. 1a,c), as occurs with non-transfected neurons. We confirmed the inability of control cortical neurons to incorporate BrdU by performing double immunolabeling with a highly specific anti-MAP2 chick antibody that shows the same specificity as NeuN together with anti-BrdU (not shown). In contrast, several NeuN-negative cells in our cultures were able to incorporate BrdU in either condition (arrowheads in Fig. 1b). A small proportion of TAg-transfected neurons showed BrdU-specific immunostaining already after 1 day post-transfection (dpt) (Fig. 1b,e), and the percentage of BrdU incorporation reached a plateau after 2 dpt, when around 70% of transfected cortical neurons incorporated BrdU (Fig. 1d,e). These observations indicate that cell cycle reentry can be induced by TAg in NeuN-positive neurons and that most of them are able to undergo S-phase.

**Figure 2:**
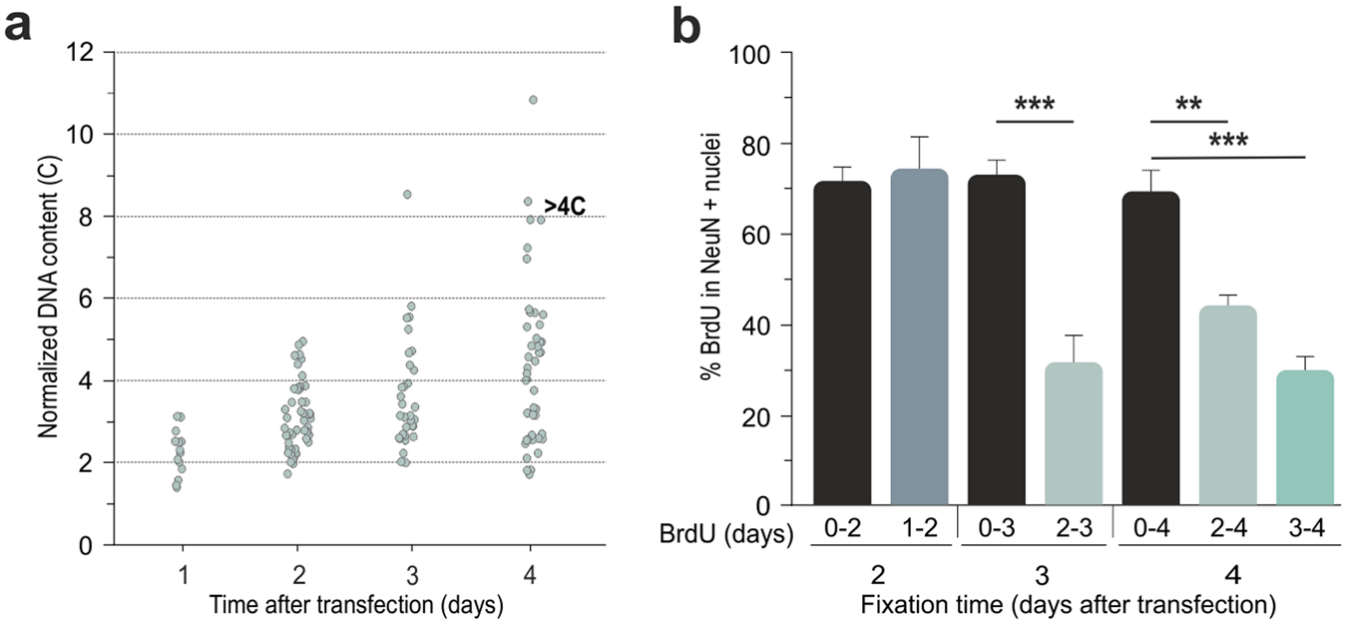
TAg-expression induces hyperploidy in cortical neurons. (**a**) DNA content in BrdU-positive, RFP/TAg-transfected cortical neurons, normalized to the average DNA content in non-transfected cortical neurons from the same microscopic field, at the indicated time points. Each dot represents the normalized DNA content for a single NeuN/BrdU-positive nucleus. (**b**) BrdU pulse-and-chase analysis in RFP/TAg-transfected cortical neurons to determine the proportion of cortical neurons incorporating BrdU at the indicated time intervals. **p < 0.01, ***p < 0.001. Error bars indicate SEM (n = 3).

As expected, BrdU incorporation in cortical neurons correlated with gradual increase of DNA levels, as evidenced by DAPI quantification in NeuN/BrdU-double positive nuclei (Fig. 2a). This analysis evidenced that some neurons acquired DNA content above 4 C, suggesting that they had entered into an endoreplicative cycle^21^. This latter view is consistent with BrdU pulse and chase experiments performed in cultures transfected with the TAg construct. This analysis indicated that around 30–40% of transfected neurons still incorporate BrdU after 2–4 dpt (Fig. 2b; 0–3 vs. 2–3: t(6) = 6.285, p < 0.001; 0–4 vs. 2–4: t(5) = 4.544, p = 0.006; 0–4 vs. 3–4: t(6) = 7.494, p < 0.001; two-tailed Student’s *t* test).

### Cell cycle reentry induces non-apoptotic/oxidative stress-independent cell death in neurons

**Figure 3:**
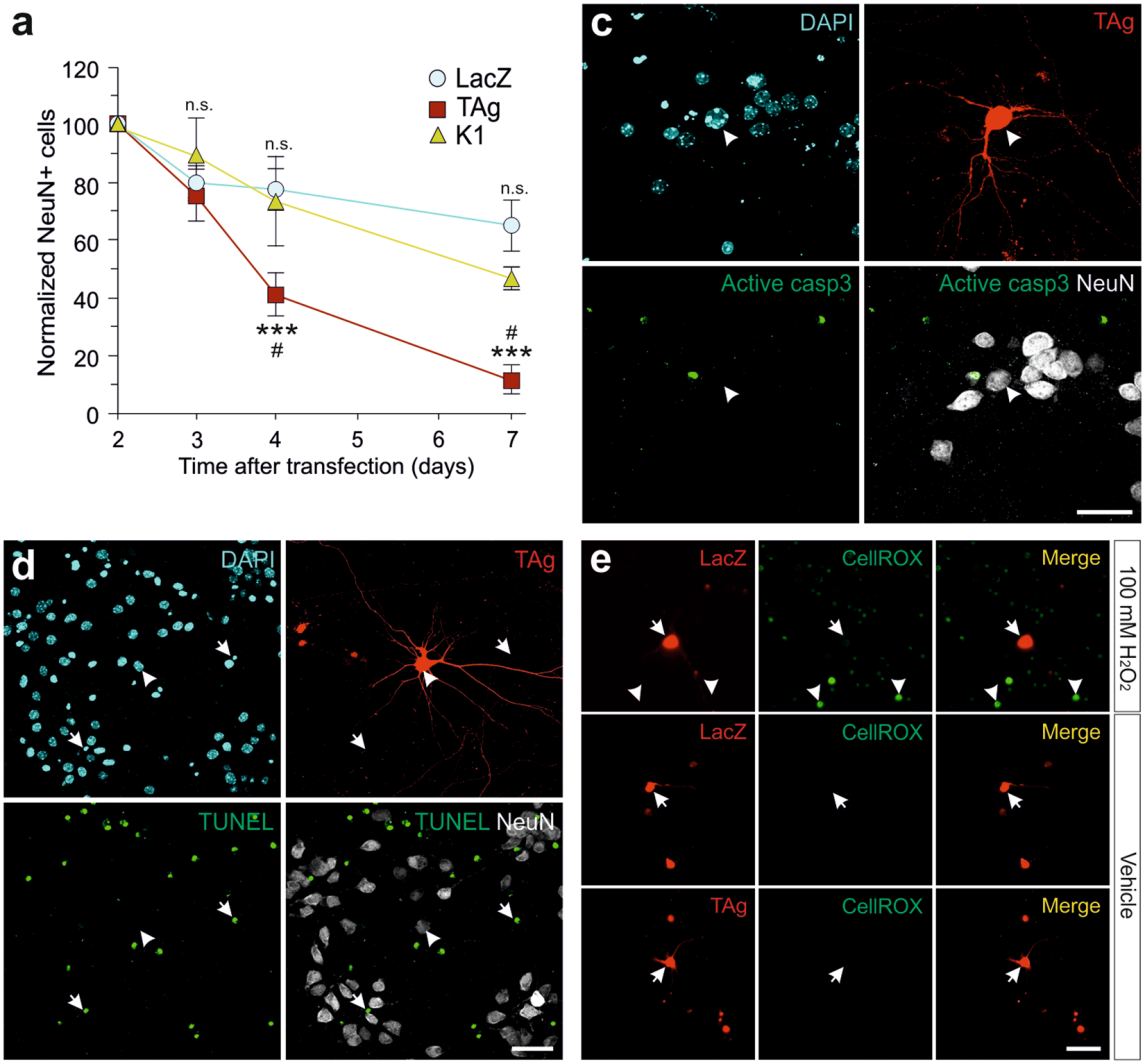
Cell cycle reentry induces non apoptotic cell death in cortical neurons, which is independent of oxidative stress. (**a**) Number of neurons co-transfected with RFP and either LacZ, TAg, or TAg K1 surviving at the indicated time points, normalized to the number of transfected neurons at 2 dpt, a stage in which the maximal number of RFP-positive neurons can be observed. ***p < 0.001 (TAg vs LacZ), ^#^p < 0.05 (TAg vs K1), n.s. non-significant (Tukey’s honestly significant difference test). (**b**) Active Caspase-3 immunostaining (green) at 7 dpt in cortical neurons (NeuN; white) co-transfected with RFP and TAg. Cell nuclei are evidenced by DAPI (blue). (**c**) TUNEL staining (green) at 3 dpt in cortical neurons (NeuN) co-transfected with RFP and TAg. Cell nuclei are evidenced by DAPI (blue). Transfected neurons (arrow) lack TUNEL staining while some non-transfected cells (arrowheads) are TUNEL-positive. (**d**) CellROX Green labeling can be observed in cortical cells treated with 100 μM H_2_O_2_ (arrowheads). In contrast, no labeling is observed under control conditions in cortical neurons co-transfected with LacZ or TAg (3 dpt). Arrows: transfected cells. **p < 0.01; ***p < 0.001. Error bars indicate SEM. Bars: 40 μm.

The fate of TAg-expressing cortical neurons was determined by counting the absolute number of RFP/NeuN-positive cells observed at different time points, normalized to the number of transfected neurons after 2 dpt (when maximal BrdU incorporation can be observed; Fig. 1e). Furthermore, at this time point the absolute number of neurons expressing RFP reaches its peak [only 66.49 ± 8.77% (n = 3) of the number of RFP/NeuN double positive cells observed at 2 dpt could be detected at 1 dpt in the LacZ/RFP-transfected cultures, while this percentage was 83.78 ± 17.2 (n = 3) in the TAg/RFP-transfected cultures]. The analysis of the survival kinetics yielded non-significant differences between TAg-transfected and LacZ-transfected neurons during the first 3 dpt. In contrast, at later time points the normalized number of TAg-transfected neurons was significantly reduced in comparison to control, LacZ-transfected neurons (Fig. 3a; days: F_(3,59)_ = 36.91, p < 0.001; transfection type: F_(2,59)_ = 14.61, p < 0.001; interaction: F_(6,59)_ = 4.18, p < 0.01; two-way ANOVA). We therefore concluded that TAg expression leads to delayed cell death. This effect depends exclusively on the capacity of TAg to favor cell cycle reentry since cortical neurons transfected with TAg K1, E107K TAg variant that cannot bind to the pRb family members^22^, could not induce BrdU incorporation (not shown), DNA levels increase (Fig. S2), and cell death compared to the control (Fig. 3a) in cortical neurons. As expected, the normalized number of TAg-transfected neurons was also significantly reduced at later time points in comparison to TAg K1-transfected neurons (Fig. 3a).

To study whether cell cycle reentry-associated cell death in cortical neurons is of apoptotic nature, TAg-transfected cultures were fixed at 2, 3, 4, and 7 dpt and co-immunostained with antibodies against NeuN and active Caspase-3. This analysis indicated that only 2 out of the 233 analyzed neurons from 2 independent experiments examined at the indicated time points were positive for active Caspase-3 (Fig. 3b), thus suggesting that TAg-expressing neurons die through a non-apoptotic mechanism. This conclusion was confirmed by terminal deoxynucleotidyl transferase dUTP nick end labeling (TUNEL) staining in TAg-transfected cortical cultures after 3, 4, and 7 dpt (Fig. 3c). TUNEL labeling was absent in all NeuN-positive neurons that were analyzed in 2 independent experiments [n = 256 (3 dpt), n = 242 (4 dpt), n = 113 (7 dpt)]. We therefore concluded that apoptosis does not participate in the death of cortical neurons that have reactivated the cell cycle in response to TAg.

Oxidative stress is known to be associated with neurodegeneration^23^. To test whether it could play a role in cell cycle reentry-associated cell death of cortical neurons, the fluorogenic probe CellROX Green was added at 4 dpt to measure oxidative stress in the TAg-transfected neurons. In contrast to hydrogen peroxide-treated cultures, in which neurons showed fluorescent CellROX Green labeling, no fluorescence signal was evident in TAg-transfected cultures (n = 168 cells from 2 independent experiments) (Fig. 3d). Therefore, TAg-associated cell death seems to be independent of oxidative stress.

### Cell cycle reentry leads to synaptic dysfunction in cortical neurons

**Figure 4:**
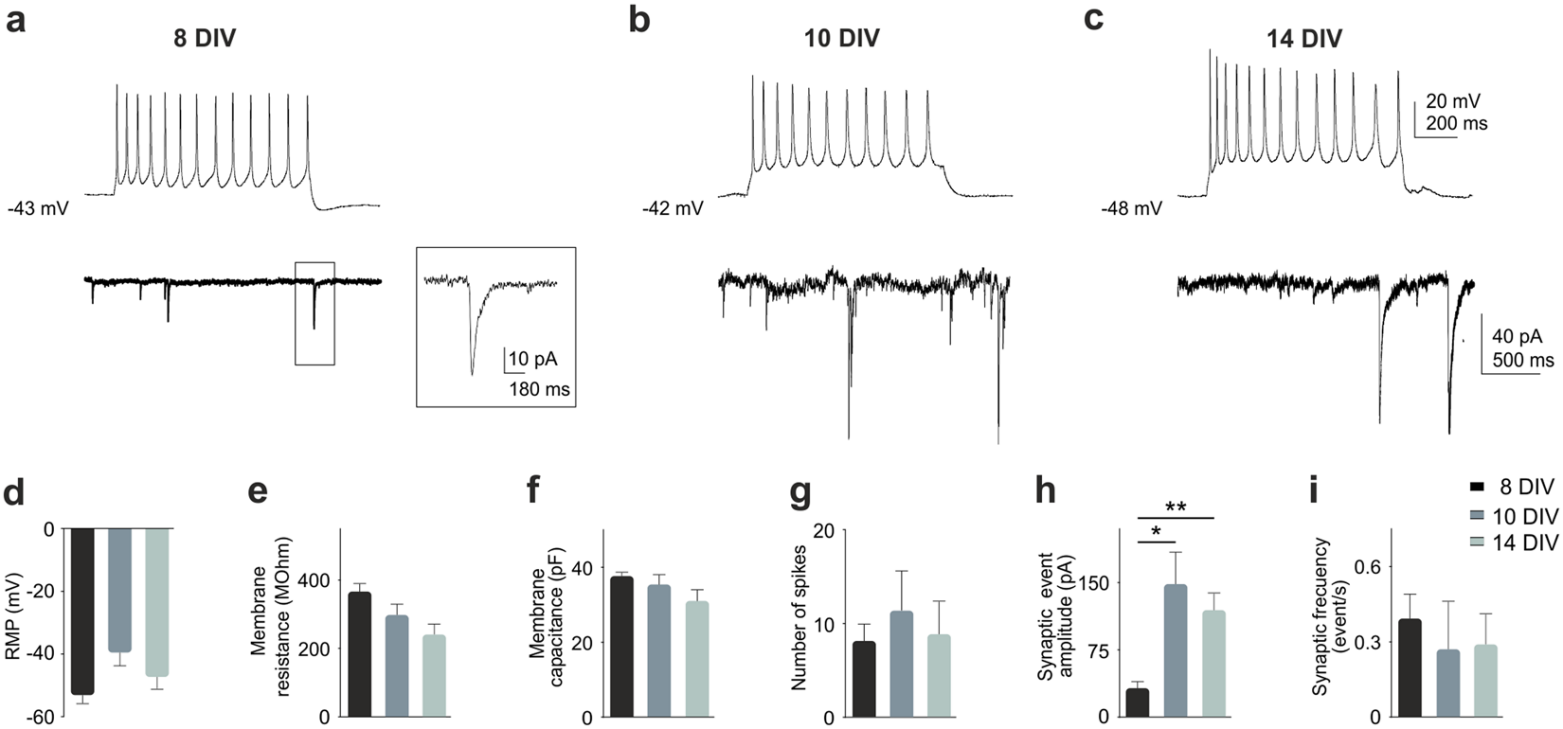
Electrophysiological profile of cortical neurons *in vitro*. (**a**–**c**) Upper traces, representative firing profiles of cells grown for 8, 10 and 14 DIV (injected current pulse 100 pA, 1 s). Bottom traces, representative traces of spontaneous synaptic currents recordings from these cells. Insert in A: high magnification of the bottom trace. (**d**–**i**) Quantification of the indicated electrophysiological parameters. Amplitude of synaptic events showed a significant increase, while the rest of parameters were unchanged at these time points. *p < 0.05; **p < 0.01. Error bars indicate SEM.

Initially, we characterized a number of electrophysiological parameters in non-transfected cortical neurons from 8 to 14 DIV, including resting membrane potential (RMP), membrane resistance (R_m_), membrane capacitance (C_m_), spontaneous synaptic activity, and action potentials (APs) generation in response to depolarizing current pulses. This analysis indicated that differentiated cortical neurons are electrically active at all analyzed time points. Neurons were able to fire APs (Fig. 4a–c, upper traces) without differences in the number of spikes (Fig. 4g), and did not show differences in passive electrophysiological properties (RMP, R_m_, or C_m_) either (Fig. 4d–f). In contrast, spontaneous synaptic transmission was improved with time (Fig. 4a–c, bottom traces), boosting the amplitude of synaptic events (Fig. 4h; 8 DIV vs. 10 DIV: t(23) = 4.199, p = 0.026; 8 DIV vs. 14 DIV: t(24) = 3.379, p = 0.007; Mann-Whitney rank sum test) without changing their frequency (Fig. 4i). This observation likely reflects the maturation of the synaptic contacts of these neurons throughout the days in culture.

**Figure 5:**
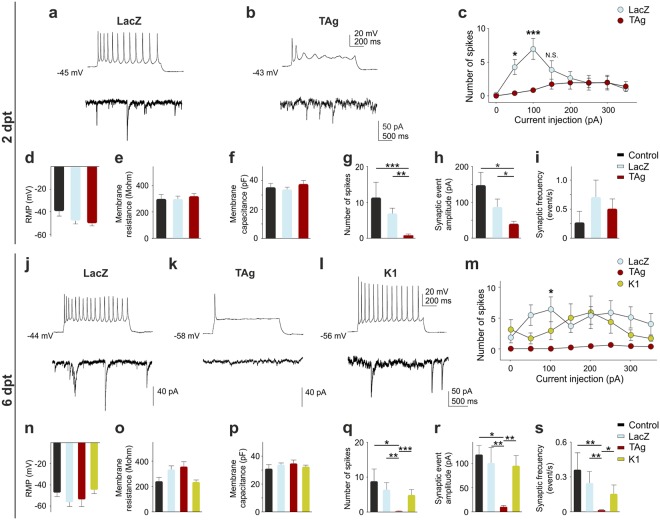
Cell cycle reentry changes firing rate and synaptic responses in cortical neurons. (**a**,**b**) Upper traces, representative firing profiles of neurons co-transfected for 2 days with RFP and either LacZ (**a**) or TAg (**b**) (injected current pulse 100 pA, 1 s). Bottom traces, representative traces of spontaneous synaptic currents from these cells. (**c**) Input–output curves for 2 dpt neurons under the indicated treatments. (**d**–**i**) Histograms showing the different parameters monitored from neurons non-transfected (Control) and co-transfected for 2 days with RFP and either LacZ or TAg. (**j**–**l**) Upper traces, representative firing profiles of neurons co-transfected for 6 days with RFP and either LacZ (**j**), TAg (**k**), or pSG5-K1 (**l**) (injected current pulse 100 pA, 1 s). Bottom traces, representative traces of spontaneous synaptic currents from these cells. (**m**) Input–output curves for 6 dpt neurons under different treatments. Note the significant decrease in excitability for TAg compared with LacZ. (**n**–**s**) Histograms showing the different parameters recorded. *p < 0.05; **p < 0.01; ***p < 0.001. Error bars indicate SEM.

A similar study was performed in cortical cultures transfected at 8 DIV with RFP along with either LacZ or TAg. Transfected neurons were recorded at 2 dpt (when maximal BrdU incorporation without cell death is observed) and 6 dpt (just before maximal cell death). This analysis indicated that RMP, R_m_, and C_m_ were not affected by TAg-dependent cell cycle reentry at any of the experimental points (Fig. 5d–f,n–p). Therefore, the capacity of TAg-expressing cells to maintain these electrophysiological parameters at normal values demonstrates the membrane integrity of these neurons.

**Figure 6:**
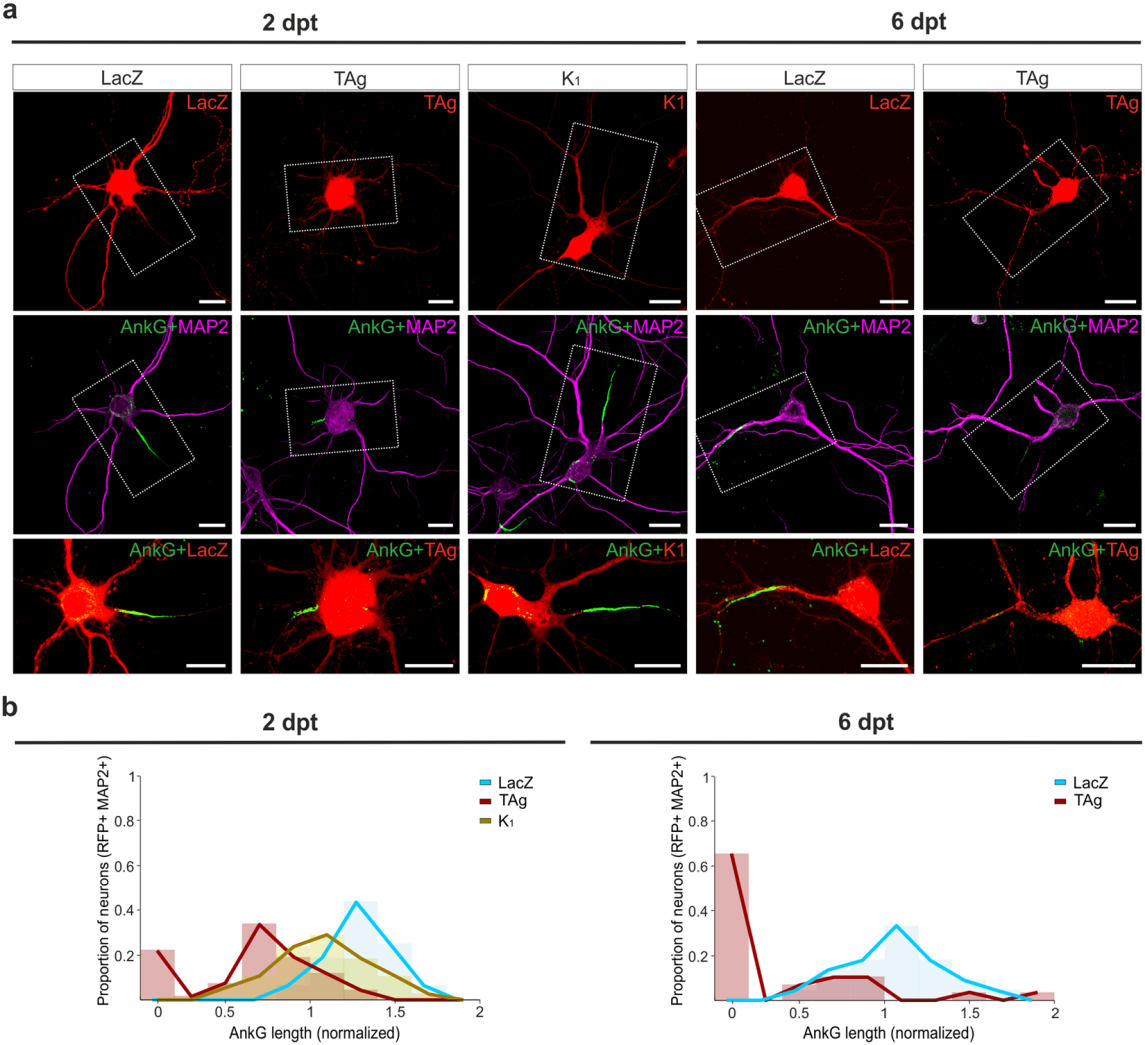
Cell cycle reentry results in progressive AIS shortening in cortical neurons. (**a**) Representative confocal images of AnkG immunostaining (green) in MAP2-positive cortical neurons (purple) at the indicated time points after transfecting LacZ, TAg, or TAg K1 (K1) (red). (**b**) Histograms depicting the proportion of transfected neurons with the indicated AIS length, normalized respectively to the average AIS length of non-transfected neurons from the same cultures. Bar: 20 µm.

The intrinsic excitability of TAg-expressing cortical neurons as well as of control, LacZ-expressing neurons was evaluated by current clamp, applying current injections to evoke action potential discharges (Fig. 5c; LacZ vs. TAg: current: F_(7,245)_ = 6.011, p < 0.001; transfection type: F_(1,245)_ = 3.559, p = 0.067; interaction: F_(7,245)_ = 6.53, p < 0.001; two-way ANOVA; and *m*; LacZ vs. TAg: current: F_(7,168)_ = 0.6555, p = 0.709, transfection type: F_(1,24)_ = 21.24, p < 0.001, interaction: F_(7,168)_ = 0.5492, p = 0.796; two-way ANOVA). Under these experimental conditions, both non-transfected and LacZ-transfected neurons were able to induce trains of APs after 2 dpt (Fig. 5g) and 6 dpt (Fig. 5q). In contrast, the firing capacity was reduced in TAg-transfected neurons as the number of spikes was significantly reduced after 2 dpt (Fig. 5g; Control vs. TAg: t(24) = 3.764, p = 0.003; LacZ vs. TAg: t(35) = 3.954, p < 0.001; Mann-Whitney rank sum test) being this effect potentiated after 6 dpt (Fig. 5q; Control vs. TAg: t(16) = 3.612, p = 0.016; LacZ vs. TAg: t(26) = 2.620, p = 0.009; Mann-Whitney rank sum test). Indeed, current injection at this latter time point could only induce a single AP in most of the TAg-transfected neurons (Fig. 5k, upper trace), whereas lipofection *per se* did not alter the APs discharge as it could clearly be observed in both non-transfected (not shown, but see Fig. 4c, upper trace) and LacZ-transfected neurons (Fig. 5j).

Additional features of neuronal activity such as the frequency and strength of synaptic responses were also evaluated. Voltage clamp recordings evidenced that TAg-transfected neurons at 2 dpt displayed spontaneous synaptic responses (Fig. 5b, lower trace), further supporting that TAg-transfected neurons are sensing surrounding neuronal activity. Indeed, no significant differences were observed in the frequency of synaptic events between TAg-transfected neurons and non-transfected and LacZ-transfected neurons (Fig. 5i). Nevertheless, the amplitude of synaptic events (Fig. 5a,b, bottom traces) in TAg-transfected neurons was significantly lower than in non-transfected or control, LacZ-transfected neurons (Fig. 5h; Control vs. TAg: t(9) = −2.872, p = 0.024; LacZ vs. TAg t(17) = −2.0017, p = 0.031; Mann-Whitney rank sum test). These effects were further enhanced after 6 dpt (Fig. 5j,k, bottom traces), when TAg-transfected neurons showed a dramatic reduction of the amplitude and frequency of synaptic responses (Fig. 5r; Control vs. TAg: t(7) = 5.363, p = 0.016; LacZ vs. TAg: t(14) = 1.912, p = 0.009; and *s;* Control vs. TAg: t(10) = 3.628, p = 0.004; LacZ vs. TAg: t(22) = 1.727, p = 0.003; Mann-Whitney rank sum test). Overall, these results indicate that TAg expression alters the excitability and synaptic features of cortical neurons. To confirm that TAg-induced cell cycle reentry caused these alterations, cortical neurons were transfected with TAg K1. Under these conditions, TAg K1-transfected cells did not show changes in the electrical parameters and synaptic responses analyzed, displaying a similar behavior to non-transfected and LacZ-transfected neurons (Fig. 5l,n–s). In addition, TAg K1-transfected neurons showed a significant improvement of the number of spikes, amplitude, and frequency of synaptic responses compared with TAg (Fig. 5q; K1 vs. TAg: t(19) = 3.554, p < 0.001; ***5r***; K1 vs. TAg: t(11) = 3.163, p = 0.002; and *5s*; K1 vs. TAg: t(15) = 1.701, p = 0.018; Mann-Whitney rank sum test).

### Reduced firing capacity induced by cell cycle reentry correlates with AIS deficiency

To explore possible mechanisms underlying the reduction of firing capacity of neurons that reactivate the cell cycle we focused on the AIS, a structure that integrates the somatodendritic synaptic input and initiates axonal action potentials^24^, and whose length reduction is known to prevent repetitive spike firing^25^. Immunostaining of the membrane scaffolding protein ankyrin-G (AnkG) was used to quantify AIS length and peak of fluorescence value (PFV)^26^ in LacZ-, TAg-, and K1-transfected neurons. The observed values of these parameters were then normalized against those from non-transfected neurons in the same microscopic fields (Fig. 6a). This analysis indicated that, at 2 dpt, both the average length of the AIS and the AIS PFV were significantly reduced in TAg-expressing neurons (Fig. S3a; LacZ vs. TAg: t(82) = 6,661, p < 0.001; and S2*c*; LacZ vs. TAg: t(82) = 2.993, p = 0.003; Mann-Whitney rank sum test). This effect depends on the capacity of TAg to induce cell cycle reentry since the AIS of cortical neurons transfected with TAg K1 was not affected (Fig. S3a; K1 vs. TAg: t(104) = 6.149, p < 0.001; and S1C; K1 vs. TAg: t(104) = 3.576, p < 0.001; Mann-Whitney rank sum test). At this time point, the reduction of the AIS length was dramatic in a small subpopulation of the TAg-expressing neurons (~20%) whereas this parameter showed a slight reduction in the rest of these neurons (Fig. 6a,b, left panels).

The length of the AIS was progressively reduced in the neurons that reactivate the cell cycle since, at 6 dpt, this structure was absent in ~65% of TAg-expressing cortical neurons that remain alive (Fig. 6a,b, right panels), whilst the AIS length value remained reduced in the rest of TAg-expressing neurons (Fig. 6b, right panels). Globally, there was a statistically significant reduction of both length of the AIS and AIS PFV (Fig. S3b; LacZ vs. TAg: t(72) = 8.259, p < 0.001; and S2*d*; LacZ vs. TAg: t(72) = 5.098, p < 0.001; Mann-Whitney rank sum test). These results suggest that the progressive reduction in the length and eventual loss of AIS participates in the reduction of firing signaling observed in TAg-expressing neurons (Fig. 5b and g vs. l,q).

### Reduced spontaneous synaptic activity induced by cell cycle reentry correlates with diminished density of PSD-95

**Figure 7:**
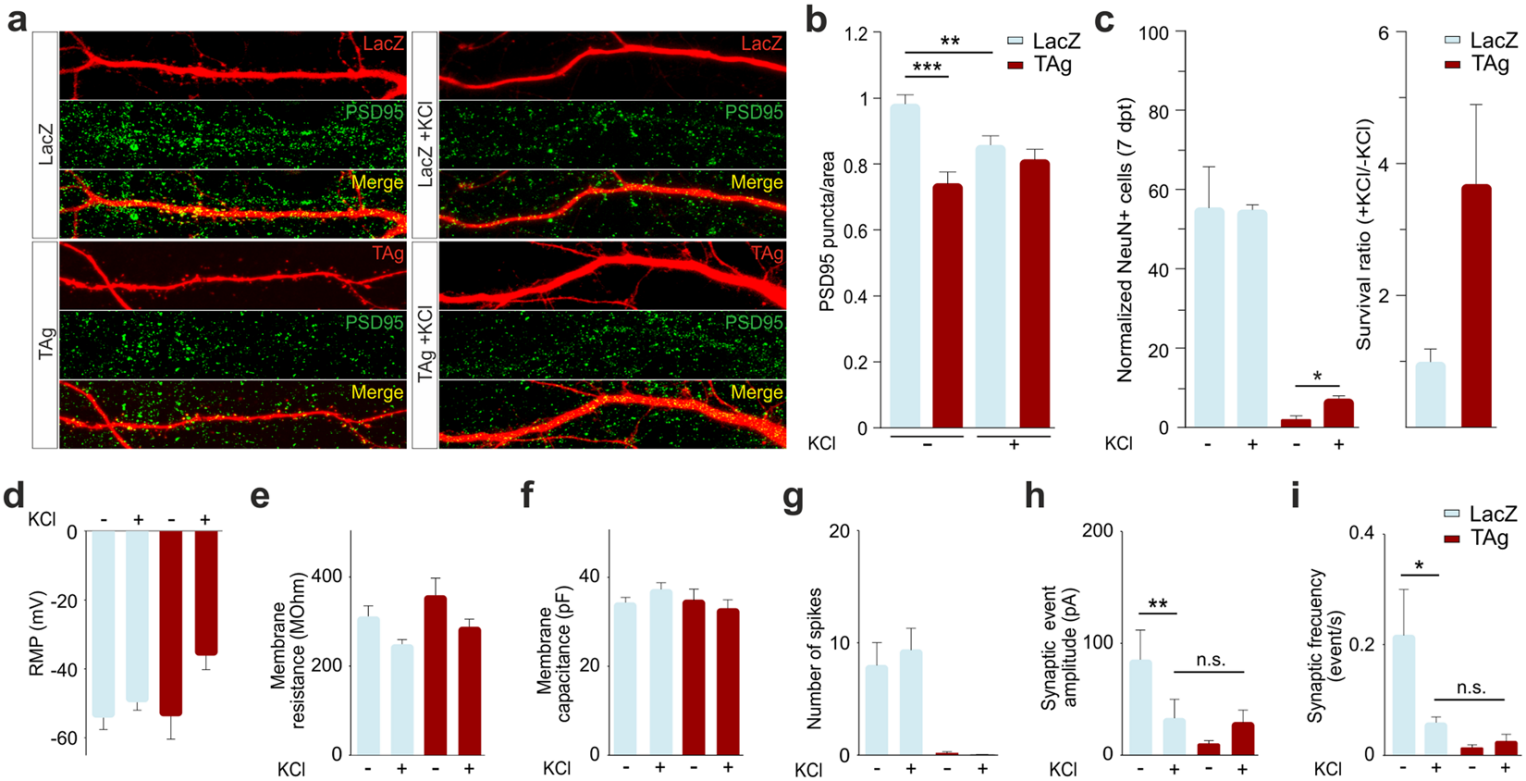
Chronic membrane depolarization maintains dendritic density of PSD-95 puncta in TAg-expressing neurons, recovers spontaneous synaptic activity, and partially prevents cell death. (**a**) Representative confocal projection images showing labeling of PSD-95 (green) in dendrites (red) of 6 dpt neurons under the indicated experimental conditions. (**b**) Number of PSD-95-specific puncta, normalized to the control situation, in cortical neurons co-transfected with RFP and either LacZ or TAg at 6 dpt. Cultures were treated with either vehicle or 15 mM KCl from 2 dpt to 6 dpt. (**c**) Left panel: Number of living neurons co-transfected with RFP and either LacZ or TAg in the presence or absence of 15 mM KCl, normalized to the number of transfected neurons at 2 dpt. Analysis was performed at 7 dpt. Right panel: survival ratio of control RFP/LacZ-transfected neurons and RFP/TAg-transfected neurons cultured in the presence versus absence of 15 mM KCl. (**d**–**i**) Histograms showing the different parameters monitored at 6 dpt in neurons under the conditions described above. *p < 0.05; **p < 0.01; ***p < 0.001. Error bars indicate SEM.

To decipher the molecular mechanism that control the reduction of spontaneous activity upon cell cycle reentry in cortical neurons we focused on PSD-95. This postsynaptic scaffold protein is required for synapse formation and function^27^. Cortical neurons were transfected with RFP plus either LacZ or TAg, and then maintained in culture until 6 dpt. At this time point, the number of PSD-95-positive puncta per dendritic area in TAg-expressing neurons that remain alive was significantly reduced when compared to LacZ-expressing neurons (Fig. 7a,b*;* LacZ -KCl vs. TAg -KCl: t(105) = 5.093, p < 0.001; two-tailed Student’s *t* test). These results suggest that reduced synaptic transmission observed in TAg-expressing cortical neurons is likely due, at least in part, to the diminished expression of components constituting the scaffolding network required for synapse formation.

### Maintenance of PSD-95 levels and partial spontaneous synaptic activity recovery and survival of TAg-expressing neurons treated with high extracellular potassium

Chronic membrane depolarization in the presence of high extracellular potassium increases neuronal excitability and regulates synaptic plasticity in cortical neurons^28,29^. This process is known to facilitate neuronal survival^30,31^.

We tested whether exposure of transfected neurons to 15 mM KCl (high extracellular potassium) from 2 dpt to 6 dpt resulted in changes in cellular excitability and synaptic parameters. LacZ-transfected neurons maintained in high potassium conditions showed a significant reduction of PSD-95-specific puncta (Fig. 7a,b; LacZ –KCl vs. LacZ + KCl: t(86) = 3.276, p = 0.002; two-tailed Student’s *t* test), a suggested mechanism of neurons to adapt their activity to the enhanced excitability^32,33^. Remarkably, the density of PSD-95-specific puncta in dendrites from TAg-expressing neurons did not differ from control neurons cultured under high extracellular potassium conditions (Fig. 7a,b), thus the molecular effects triggered by a sustained increase of excitability prevents the loss of PSD-95 puncta induced by cell cycle reentry.

To verify whether chronic exposure to high potassium can also restore the electrical properties of TAg-expressing cortical neurons, we performed electrophysiological recordings. The exposure of differentiated cortical cultures to high potassium did not altered the resting membrane potential (RMP), Rm or Cm of the neurons (Fig. 7d–f). The amplitude of spontaneous events was reduced in LacZ-expressing cortical neurons maintained in the presence of high potassium (Fig. 7h; t(27) = 1.697, p = 0.002; Mann-Whitney rank sum test), an observation consistent with the decrease of PSD-95-specific puncta triggered by this treatment (Fig. 7a,b). In contrast, the spontaneous event amplitude showed a marked, although not statistically significant increase in TAg-expressing cortical neurons cultured with high potassium, reaching similar levels to those observed in LacZ-transfected neurons maintained under this condition (Fig. 7h). High potassium treatment also induced a statistically-significant reduction of synaptic event frequency in LacZ-expressing cortical neurons (Fig. 7i; t(31) = 1.967, p = 0.038; Mann-Whitney rank sum test). As with the spontaneous event amplitude, this parameter also showed a moderate but not statistically significant increase in TAg-transfected neurons, reaching similar levels to those observed in LacZ-transfected neurons (Fig. 7i). In contrast, the number of spikes in response to depolarizing stimuli was not improved by high potassium treatment (Fig. 7g), suggesting that enhanced activity did not recover the reduced ability of TAg-expressing neurons to fire trains of APs. Overall, these results indicate that electrical activity facilitation results in both maintenance of PSD-95 and a slight enhancement of spontaneous synaptic activity in neurons that reactivate the cell cycle. The observed enhancement of spontaneous synaptic activity in TAg-transfected neurons treated from 2 dpt to 6 dpt with high extracellular potassium correlated with partial cell death rescue (Fig. 7c; TAg + KCl vs. TAg + KCl: t(2) = −7.280, p = 0.018; two-tailed Student’s *t* test), thus indicating that synaptic dysfunction in hyperploid cortical neurons could participate in their delayed death observed in AD.

## Discussion

We have shown that TAg expression results in DNA synthesis and hyperploidy in around 70% of transfected neurons. This is followed by a gradual decrease of neuronal viability starting 3 days after transfection, which is independent of caspase-3-mediated apoptosis and oxidative stress. TAg-expressing neurons maintain a normal membrane potential and are electrically active, although neuronal cell cycle reentry gradually leads to reduced ability of spike shooting upon depolarization and diminished spontaneous synaptic inputs, the latter correlating with reduced PSD-95-specific puncta in dendrites. These effects are specific for cell cycle progression since the E107K mutant form of TAg could not induce cell cycle reentry in neurons, resulting in both absence of cell death and similar levels of synaptic activity to non-transfected cells. We have also shown that chronic membrane depolarization in TAg-expressing neurons stimulated spontaneous synaptic activity and prevented PSD-95 additional loss, which correlated with neuronal survival.

Our study demonstrates that most cortical neurons are susceptible to reactivate the cell cycle in response to TAg expression, based on BrdU incorporation and increased DNA content in NeuN-positive cells. The readiness of most cortical neurons to undergo S-phase progression supports the hypothesis that neurons are not true postmitotic cells and that, under certain circumstances, can undergo cell cycle progression^34^.

In our study, neuronal identity was evaluated using the neuron-specific marker NeuN^20^ as well as electrophysiological recordings. In our hands, NeuN-positive cells do not incorporate BrdU under control conditions. Therefore, this is an optimal marker for differentiated neurons when studying cell cycle reentry.

We have found that cell cycle reentry in cortical neurons is a slow process that requires at least 48 h to get maximal BrdU incorporation, a time period longer than the standard G1 length observed in mouse cortical precursors, whose duration has been quantified in the range of 3.2–12.4 h^35^. This suggests that, in order to reactivate the cell cycle, neurons need to implement a sequential network of signals. Future studies will determine the actual mechanism involved in TAg-dependent cell cycle reentry in cortical neurons.

Cortical neurons showed variable capacity to undergo hyperploidization, with a subpopulation capable to enter into an endoreplicative cycle. In addition, ~30% of cortical neurons seem to be refractory to undergo DNA synthesis. These neurons are likely to die following similar kinetics to those of hyperploid neurons since its proportion does not change with time (Fig. 1e), even though neuronal viability dramatically drops from 3 dpt on (Fig. 3a). Otherwise, we would expect a significant drop in the proportion of BrdU-positive neurons by 4 dpt. Altogether, these results are consistent with the diversity of deleterious cell cycle responses in differentiated neurons, including abortive cell cycle reentry and non-abortive cell cycle reentry leading to tetraploidy/hyperploidy^34^. The variety of outcomes in response to cell cycle reentry could partially explain the diversity of genomic dose alterations observed in AD neurons^36^.

After cell cycle withdrawal, neurons use the cell cycle machinery to regulate differentiated functions including synaptic plasticity^37,38^. This has led to the hypothesis that these effector pathways might put neurons at risk of erroneously converting signals derived from synaptic plasticity into cell cycle reentry and subsequent neuronal death^39^. In our study, we have provided evidence for an alternative view that may complement this hypothesis since forced cell cycle reentry can also lead to synaptic dysfunction.

Our data show that cell cycle reentry induced alterations in spontaneous synaptic activity, which correlate with reduced levels of PSD-95, a relevant scaffold protein enriched at glutamatergic synapses and necessary for synapse formation and functioning^27^. PSD-95 concentrates and organizes neurotransmitter receptors and signaling molecules in the postsynaptic button. Therefore, the diminished amplitude of the spontaneous synaptic events observed in these cells could correlate with a reduction of AMPA or NMDA receptors at the synapse. In addition, we have demonstrated that the reduction of spike generation in TAg-expressing neurons correlates with AIS shortening, which eventually results in full AIS depletion. Interestingly, AIS shortening is associated with dampened excitability in multiple spike firing^25^, an alteration that can be observed in the TAg-transfected neurons (Fig. 5j, upper panel). Moreover, AIS shortening represents a remarkable neuronal alteration detected in murine models of Alzheimer^40,41^, in which the expression of exogenous AnkG improves their cognitive performance^42^. Considering that the G1/S regulator Cdk2 can phosphorylate the Kvβ2 auxiliary subunit of the Kv1 channel^43^, it is conceivable that an obstruction in the targeting of the Kv1 channel in the AIS could be occurring, which may also alter spike firing^44^.

Although future studies are required to uncover the molecular mechanisms involved in the synaptic dysfunction driven by cell cycle reentry, we have found that this type of synaptic dysfunction derives from the capacity of TAg to trigger cell cycle reentry in neurons. Indeed, a mutation within its Rb binding site, which prevents the capacity of this viral molecule to trigger cell cycle progression^45^, does not interfere with the synaptic function either. This indicates that the observed synaptic dysfunction in hyperploid neurons is not due to a hypothetical side effect of the TAg protein.

Cell cycle reentry in cortical neurons leads to non-apoptotic cell death independent of oxidative stress, which begins when synaptic dysfunction is already evident. These results are consistent with the neuronal health hypothesis^46^, which postulates that the physiological state of a neuron is positively influenced by synaptic activity and the reduction of the latter leads to cell death^47^.

In our study, cell cycle reentry in neurons does not lead to apoptosis. This contrasts with a number of studies demonstrating that this process results in apoptotic cell death^3,48^. The lack of apoptotic response in TAg-expressing cortical neurons is consistent with the capacity of TAg to inhibit p53^49^, a known tumor suppressor that triggers apoptosis in neurons that reactivate the cell cycle^50^. A hallmark of AD is its association with mutated p53^51^. Therefore, it is tempting to speculate that the existence of p53 mutations may facilitate the accumulation of hyperploid neurons in the Alzheimer brain. The known accumulation of p53 mutations with age^52^ might also be responsible for the observed age-associated increase of neuronal tetraploidy in the human brain^7^.

Our observation that cell cycle reentry induces non-apoptotic cell death in cortical neurons is reminiscent of the pathological situation in AD. Indeed, no direct proof exists for the participation of apoptosis in this neuropathological condition^53^, and the exact mechanisms triggering neuronal death is a question of debate^54^. Neurons becoming hyperploid in AD have been shown to undergo cell death at later stages of disease progression^5,8–10^, which is reminiscent of what can be observed in our cultures.

During development, some migrating neuroblasts reactivate the cell cycle and become tetraploid^55,56^. These cells differentiate and survive as tetraploid neurons, and remain functionally active in the adult brain^56^. Tetraploidization affecting migrating neuroblasts, which are synaptically inmature, contrasts with hyperploidization in fully differentiated neurons, which leads to synaptic dysfunction and delayed cell death.

We have provided evidence that the deficits of spontaneous synaptic activity detected in TAg-expressing cortical neurons can be prevented by chronic membrane depolarization with high extracellular potassium. This observation is consistent with the known capacity of membrane depolarization to regulate synaptic plasticity in cortical neurons^28,29^. Indeed, the presence of high extracellular potassium in our cultures resulted in the enhancement of the amplitude to control levels and a modest increase of frequency of spontaneous synaptic events in TAg-expressing neurons. In contrast, high extracellular potassium did not recover the capacity of these neurons to fire action potentials. This finding is consistent with the known shortening of AIS upon KCl-dependent elevated activity^25^.

High extracellular potassium-induced spontaneous synaptic improvement in TAg-expressing cortical neurons potentiated cell survival. This suggests that the electrically-active environment in the brain could be responsible, at least partially, for the observed long-range survival of hyperploid neurons *in vivo*^5,8–10^. We suggest that the reduced capacity of hyperploid neurons to fire APs, which cannot be reverted by high potassium, could perturb the neuronal networks where they are integrated, a mechanism that may account for the cognitive loss observed at initial stages of AD, when neuronal death is not yet evident. This hypothesis is consistent with a recent study modeling cognitive deficits following neurodegenerative diseases^57^. This study demonstrates that synaptic damage leads to human-like mistakes in convolutional neural networks and that the accuracy obtained in the simulation largely depends on the weight of the affected neuronal connections. Therefore, major functional alterations are expected if a relatively small proportion of neurons constituting important nodes of the neural network remains alive while having synaptic damage due to cell cycle reentry.

**Figure 8:**
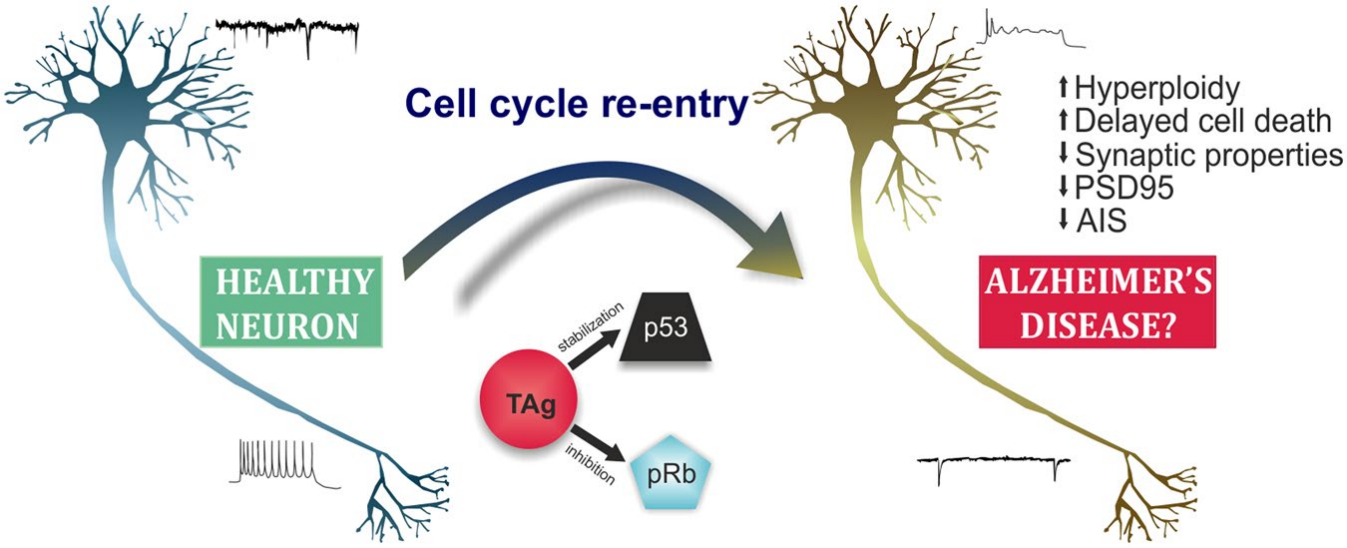
Scheme summarizing the main results from this study. TAg induces cell cycle reentry in healthy neurons by blocking pRb protein. The capacity of TAg to inhibit p53 might help to prevent apoptosis in neurons that undergo cell cycle reentry, which nevertheless undergo delayed non-apoptotic cell death. Cell cycle reentry elicits functional changes in neurons, which might contribute to cognitive impairment and neuronal death susceptibility as observed in AD.

This study has defined an *in vitro* system for the analysis of the effects of cell cycle reentry and hyperploidization associated with p53 inhibition in neurons. To this aim, we have focused on SV40 T antigen, which has previously shown to trigger cell cycle reentry in brain neurons associated with hallmarks of AD^13^. In addition, we have shown that TAg-induced cell cycle reentry in neurons results in synaptic dysfunction followed by delayed cell death, as occurs in hyperploid neurons in the AD brain^10^. Therefore, this *in vitro* system is optimal for studying early pathophysiological events occurring in AD as well as downstream degenerative processes taking place in AD-affected neurons (Fig. 8).

## Methods

### Ethics statement

All of the procedures for handling and sacrificing animals were approved by the CSIC Ethics Committee in accordance with the EU guidelines for the care and use of laboratory animals.

### Plasmids

The pSG5 Large T (Addgene plasmid #9053) and pSG5 Large T K1 (Addgene plasmid #9055) vectors expressing TAg and the TAg K1 mutant^22^, were a gift from William Hahn (Dana-Farber Cancer Institute). The pcDNA6/V5-His/LacZ was purchased from Invitrogen. The pRFPRNAiC vector expressing red fluorescent protein (RFP), provided by Stuart Wilson (University of Sheffield, UK), was previously described^58^. The pEGFPN1 vector expressing enhanced green fluorescent protein (EGFP) was obtained from Clontech.

### Antibodies

The anti-BrdU rat monoclonal antibody (mAb) [BU1/75 (ICR1)] (AbDSerotec, RRID: AB_323427) was used at 1:200 dilution. The anti-SV40 T Ag (Pab 101) mouse mAb (Santa Cruz Biotechnology) was used at 1/200 dilution. The anti-NeuN mouse mAb A60 (Millipore, RRID: AB_2298772) was diluted 1:1000. The anti-MAP2 chicken antibody ab5392 (Abcam, RRID: AB_2138153) was diluted 1:15000. The anti-cleaved Caspase-3 rabbit antibody (Cell Signaling Technology, RRID: AB_2341188) was used at 1:400 dilution. The anti-GFP rabbit polyclonal serum [A6455] (Invitrogen, RRID: AB_221570) was used at 1:500 dilution. The anti-Ankyrin-G mouse antibody [N106/36] (NeuroMab, RRID: AB_10673030) was diluted at 1:150. The anti-PSD-95 mouse mAb [7E3-1B8] (Abcam, RRID: AB_221570) was diluted 1:500.

### Tunel

TUNEL was performed using the *In Situ* Cell Death Detection Kit, Fluorescein (Sigma Aldrich) following the specifications of the manufacturer.

### Oxidative stress analysis

CellROX Green Reagent (Thermo Fisher Scientific) was used at 5 μM for oxidative stress detection as indicated by the manufacturer.

### Cortical cultures

Primary cortical neurons derived from CD-1 mouse embryos of unknown sex (Charles River) were harvested from embryonic day 16.5–17.5, staged as previously described^59^. Pups were decapitated and brains were placed in cold Hanks’ balanced salt solution (HBSS). Meninges were then removed and the cerebral cortices were carefully dissected. Cortices were diced and incubated for 18 min at 37 °C in HBSS containing 2.5 mg/ml trypsin (Gibco) and 1 mg/ml DNAse (Roche). Cortices were dissociated by passing them through the enlarged bore of a pasteur pipette, and then Dulbecco’s modified Eagle medium (DMEM) (Thermofisher) containing 10% fetal bovine serum (FBS) (Life technologies) was added for trypsin inactivation. Dissociated cells were centrifuged at 1,050 rpm for 5 min at room temperature (RT) and the pellet was re-suspended in DMEM/FBS for counting and plating. Neurons were plated at a density of 25.000 (AIS and PSD-95 studies) or 85.000 cells/cm^2^ (rest of analyses) on coverslips (Menzel Glässer) previously coated with 0.5 mg/ml polyornithine (Sigma-Aldrich) and incubated in a humidified atmosphere containing 5% CO_2_ at 37 °C. Two hours later, Neurobasal medium supplemented with B27 (Thermo Fisher), penicillin-streptomycin (25 U/ml) (Gibco) and GlutaMAX (Gibco) was added. Half of the culture medium was exchanged by fresh medium every 3–4 days. For “pulse and chase” BrdU experiments, cortical cultures were incubated with BrdU (5 µg/ml) from the beginning of the experiment, while in other cultures BrdU was added at later time points. For chronic membrane depolarization, KCl (Merck) was added to a final concentration of 15 mM after 2 dpt. KCl concentrations higher than 15 mM resulted in neuronal death (not shown).

### Lipofection

Cortical neurons differentiated for 6–8 DIV as described above were transferred to fresh Neurobasal medium lacking antibiotics, and transfected with DNA/Lipofectamine 2000 (Invitrogen) following the indications of the manufacturer (1 μg DNA/1 μl Lipofectamine 2000). pSG5 Large T, pSG5 Large T K1, or pcDNA6/V5-His/LacZ DNA was always combined with pRFPRNAiC or pEGFPN1 DNA at a 19:1 ratio. After incubating the neurons for 2 h at 37 °C, transfection medium was exchanged by fresh Neurobasal medium containing BrdU (5 µg/ml). Neurons were then maintained in culture for 1 to 7 days. BrdU incorporation levels in response to TAg expression were identical regardless of the differentiation time (6–8 days).

### Immunocytochemistry

Cortical cultures were fixed for 15 min with 4% paraformaldehyde (PFA) at RT, and permeabilized for 30 min with phosphate buffered saline (PBS) containing 0.05% Triton X-100 (Sigma-Aldrich) (PBTx). For BrdU inmunolabeling, DNA was denatured for 30 min at RT with 2 N HCl/0.33× PBS, and then neutralized with three 15-min washes with 0.1 M sodium borate, pH 8.9, and a wash of 5 min with PBTx. Cultures were then incubated for 30 min at RT with PBTx containing 10% FBS to block antibody unspecific binding, followed by a 1-h incubation at RT with PBTx-1% FBS and the appropriate primary antibodies. After 4 washes in PBTx, cultures were incubated for 1 h at RT in PBTx containing 1% FCS and a 1/1000 dilution of Alexa Fluor 488 Goat Anti-Rat IgG (H + L) (Life Technologies, RRID: AB_2534074), Alexa Fluor 488 Goat Anti-Mouse IgG (H + L) (Life Technologies, RRID: AB_2534088), Alexa Fluor 488-conjugated Affinipure Goat Anti-Rabbit IgG (H + L) (Jackson Immunoresearch, RRID: AB_2535792), 1/1,000 dilution of Alexa Fluor 594 Goat Anti-Rabbit IgG (H + L) (Life Technologies, RRID: AB_2534095), 1/1000 dilution of Alexa Fluor 647 Goat Anti-Chicken IgY (H + L) (Life Technologies, RRID: AB_2535866) or 1/1000 dilution of Alexa 647 Donkey Anti-Mouse IgG (H + L) (Life Technologies, RRID: AB_162542). After 4 additional washes as above, nuclear labeling was performed using PBS containg 100 ng/ml 4′,6-diamidino-2-phenylindole (DAPI) (Sigma-Aldrich) and the preparations were mounted in glycerol (Panreac)/PBS (1:1).

### Slide-based cytometry (SBC)

DNA quantification was performed by measuring DAPI intensity levels in lipofected neurons. To this aim, cortical cultures were fixed for 15 min with 4% PFA, immunostained for NeuN (using Alexa Fluor 647 to avoid overlapping with DAPI signal), and labeled for 1 min with a 100 ng/ml DAPI solution prepared in PBS. Cultures were washed with PBS, and then mounted in glycerol/PBS (1:1). DAPI labeling in NeuN-positive cells was recorded under linear conditions with a Nikon E80i microscope equipped with a DXM 1200 digital camera (Nikon), using a 40x magnification objective. The integral density of DAPI, obtained as arbitrary units, was quantified using ImageJ software (National Institute of Health, RRID: SCR_003070) and at least 30 neurons were analyzed for each experimental point. Normalization of the intensity values with control NeuN-positive cells (not lipofected from the same microscopic field) was made. Neurons were considered hyperploid when the DNA amount was around 4 C or higher.

### Electrophysiological recordings

Electrophysiological experiments were performed in cultured neurons grown for 8, 10, or 14 DIV (0, 2, or 6 dpt) on glass coverslips using patch-clamp whole-cell configuration. An average of 12 neurons was recorded per experimental condition. Cells were visualized with an Olympus BX50WI microscope coupled with a 40x water immersion objective and infrared-DIC optics. The extracellular medium contained (in mM): 124 NaCl, 2.69 KCl, 1.25 KH_2_PO_4_, 2 MgSO_4_, 26 NaHCO_3_, 2 CaCl_2_, and 10 glucose, and was gassed with 95% O_2_/5% CO_2_ (pH = 7.3). The patch pipette contained (in mM): 130 K-gluconate, 10 NaCl, 1 EGTA, 0.133 CaCl_2_, 2 MgCl_2_, 10 HEPES, 3.5 MgATP, 1 NaGTP (pH = 7.4). The pipette resistance varied between 3 and 5 MΩ. Experiments were performed at 37 °C.

Resting membrane potential was estimated in whole-cell current clamp configuration at I = 0 immediately after membrane rupture. APs were evoked by depolarizing current pulses (50 pA steps, 1 s). Spontaneous synaptic transmission was recorded by clamping cells at −70 mV. Currents were obtained with PC-ONE amplifier (Dagan Corporation). Series and input resistances were monitored throughout the experiment using a −5 mV pulse. Recordings where access resistance changed >20% during the experiment were discarded from the analysis. Signals were fed to a Pentium-based PC through a DigiData 1440 interface board (Molecular Devices). The pCLAMP 10.2 software (Molecular Devices, RRID: SCR_011323) was used for data display, acquisition, and storage. Electrophysiological recordings in neurons cultured in the presence of KCl were performed under standard buffer composition.

### Neuron survival analysis

Images were acquired with a 40x objective using a TCS SP5 confocal microscope (Leica). Neuronal cell counts were performed in randomly selected fields of 385 × 385 µm. For survival analyses, the mean (n = 3) of the absolute number of RFP/NeuN-positive cells per coverslip at each time point and condition was normalized with respect to the mean obtained at 2 dpt. Then, the mean ± SEM of these ratios was estimated from 10 independent experiments. As an average, a range of 700–900 RFP/NeuN-transfected cells was present in each coverslip at 2 dpt.

### PSD-95 measurement

Images were acquired with a 40x objective using a TCS SP5 confocal microscope (Leica). The number of PSD-95-immunopositive clusters was quantified using the ImageJ software in randomly selected dendrites. Values are presented as number of PSD-95 puncta per unit of area. At least 40 cells from 2 different experiments were quantified for this analysis.

### AIS measurement

The length and peak of fluorescence intensity of the AIS was evaluated by AnkG-specific immunohistochemistry. Images of 1024 × 1024 pixels in z stacks with 0.5-μm step-size were acquired on a Leica TCS SP5 confocal microscope with a 63 × 1.4 IMM oil objective, and a maximum Z-projection was obtained. Custom-written functions were developed in ImageJ based on previously described Matlab (Mathworks) scripts^25,26^. AnkG label was first somoothed using a Gaussian Blur 1 filter, thresholded to extract the background, and converted into a binary image. Then the image was processed with open-close functions to remove discontinuities and a 1 pixel wide ‘AIS skeleton’ was obtained. We manually extended the line from MAP2 labeling in the soma through to the end of AnkG labeling in the axon. Fluorescence intensity values along the drawn line were averaged over 3 × 3 pixel around the pixel of interest (1 pixel = 0.24 µm) and smoothed using a simple moving average from BAR plugin (≈2 µm). These values were normalized between 0 (minimum smoothed fluorescence) and 1 (maximum smoothed fluorescence) using the following formula: y_2_[i] = (y_1_[i] − min)/(max − min), where y_1_ is the original smoothed fluorescence value and y_2_ the normalized one. AIS start and end position were obtained when the normalized profile surpasses and declines to 0.33 criteria, as previously described^26^. AnkG peak of fluorescence value (PFV) was obtained before intensity normalization. AIS length and PFV were normalized to values in adjacent non-transfected neurons. The total number of transfected neurons analyzed in all 4 different experiments was 196.

### Experimental design and statistical analysis

The normality test was performed before applying statistical comparisons, which were made using non parametric Mann-Whitney rank sum test, parametric ANOVA followed by Tukey’s honestly significant difference or Bonferroni post hoc test and unpaired Student’s *t* test as deemed appropriate using SigmaPlot 13.0 (Systat Software) or MATLAB (MathWorks). Quantitative data are expressed as mean ± standard error of the mean (SEM) from at least three independent experiments. When a statistical test was used, the precise p value and the test employed are reported in the text and/or figures legends. Statistically significant differences were established with *p < 0.05, **p < 0.01 and ***p < 0.001.

## Electronic supplementary material

Supplementary Information

## Data Availability

The datasets generated during and/or analyzed during the current study are available from the corresponding author on reasonable request.
