# Supplementary material for: Cell cycle reentry triggers hyperploidization and synaptic dysfunction followed by delayed cell death in differentiated cortical neurons
Source: Sci Rep. 2018 Sep 25;8:14316. doi: 10.1038/s41598-018-32708-4 (PMC6156334; doi:10.1038/s41598-018-32708-4)
Supplement: Supplementary file 1 — Supplementary Information [file 41598_2018_32708_MOESM1_ESM.pdf]

SUPLEMENTARY INFORMATION

Cell cycle reentry triggers hyperploidization and synaptic dysfunction followed by delayed cell death in differentiated cortical neurons

Estíbaliz Barrio-Alonso[1], Alicia Hernández-Vivanco[2], Chaska C. Walton[1], Gertrudis Perea[2], and José M. Frade[1]

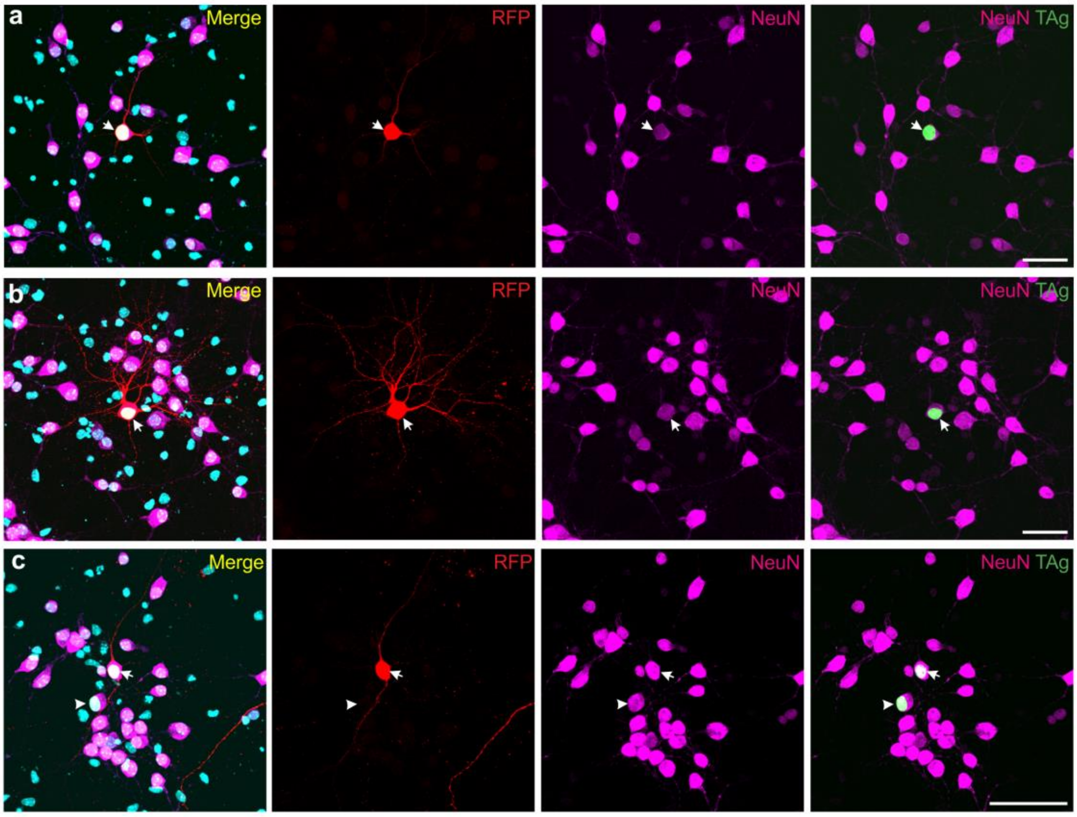

Figure S1. All RFP-positive neurons express TAg in RFP/TAg double transfected cultures. (**a-c**) Examples of RFP/TAg-transfected cortical neurons (arrows) after 3 dpt, inmunolabeled with antibodies against NeuN (purple) and TAg (green). DAPI is shown in blue. All RFP-positive neurons co-express TAg. Note that TAg can occasionally be detected in RFP-negative neurons (arrowhead). Bars: 40 μm.

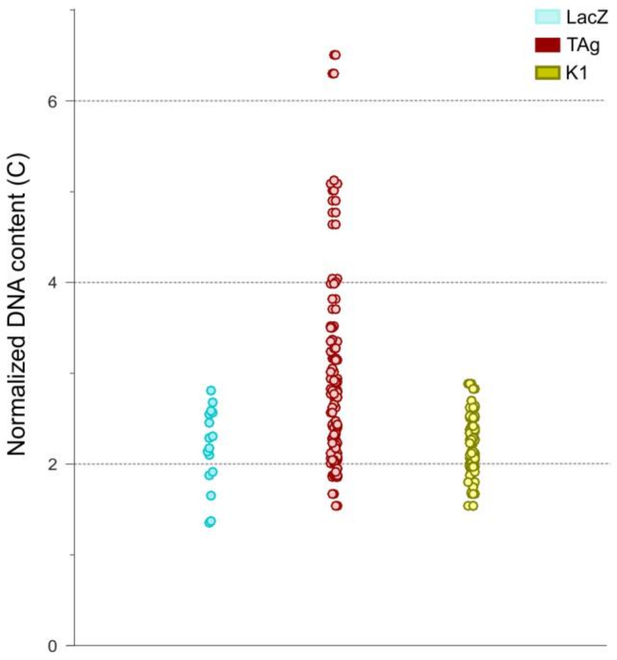

Figure S2. TAg K1-expression cannot induce hyperploidy in cortical neurons.

DNA content in RFP-positive LacZ-, TAg-, or TAg K1-transfected cortical neurons, normalized to the average DNA content in non-transfected cortical neurons from the same microscopic field, at 2dpt. Each dot represents the normalized DNA content for a single NeuN-positive nucleus.

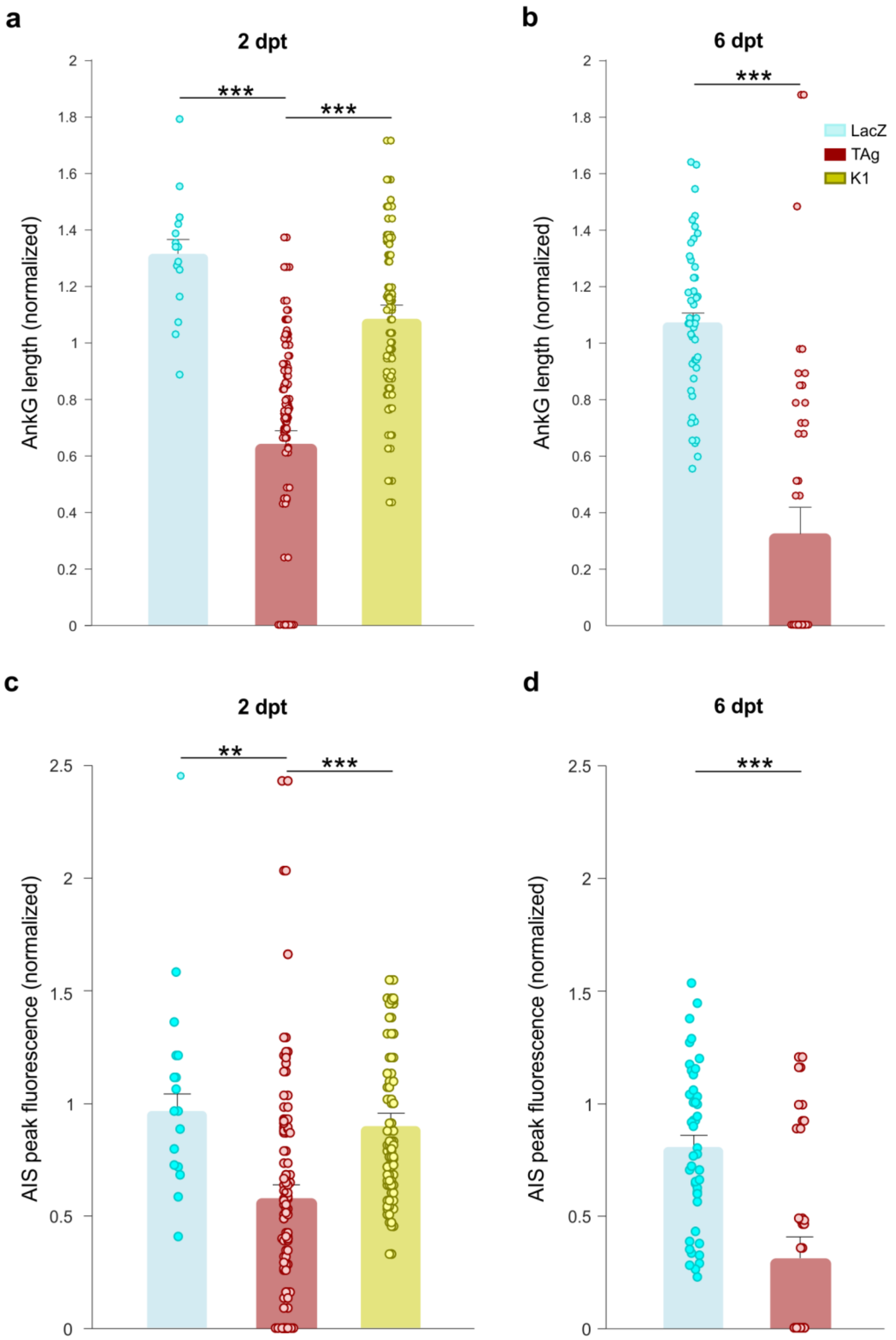

Figure S3. Hyperploidy results in progressive AIS shortening in cortical neurons. (**a-b**) Average AIS length of transfected neurons, normalized respectively to the average AIS length of non-transfected neurons from the same cultures, at 2 dpt (**a**) and 6 dpt (**b**).

Each dot represents the normalized AIS length for a single transfected-positive nucleus. (**c**-**d**) Average AIS PFV of transfected neurons, normalized respectively to the AIS PFV of non-transfected neurons from the same cultures, at 2 dpt (**c**) and 6 dpt (**d**). Each dot represents the normalized AIS PFV for a single transfected-positive nucleus. *$p<0.05$; **$p<0.01$; ***$p< 0.001$. Error bars indicate SEM.
